# Facet joint disturbance induced by miniscrews in plated cervical laminoplasty: Dose it influence the clinical and radiologic outcomes?

**DOI:** 10.1097/MD.0000000000004666

**Published:** 2016-09-23

**Authors:** Hua Chen, Huibo Li, Beiyu Wang, Tao Li, Quan Gong, Yueming Song, Hao Liu

**Affiliations:** Department of Orthopedics, West China Hospital, Sichuan University, Chengdu, Sichuan, P.R. China.

**Keywords:** axial symptoms, facet joints disturbance, laminoplasty, miniplate, miniscrews, neurological recovery, spinal canal expansion

## Abstract

A retrospective cohort study. Plated cervical laminoplasty is an increasingly common technique. A unique facet joint disturbance induced by lateral mass miniscrews penetrating articular surface was noticed. Facet joints are important to maintain cervical spine stability and kinetic balance. Whether this facet joint disturbance could affect clinical and radiologic results is still unknown. The objective of this study is to investigate the clinical and radiologic outcomes of patients with facet joints disturbance induced by miniscrews in plated cervical laminoplasty.

A total of 105 patients who underwent cervical laminoplasty with miniplate fixation between May 2010 and February 2014 were comprised. Postoperative CT images were used to identify whether facet joints destroyed by miniscrews. According to facet joints destroyed number, all the patients were divided into: group A (none facet joint destroyed), group B (1–2 facet joints destroyed), and group C (≥3 facet joints destroyed). Clinical data (JOA, VAS, and NDI scores), radiologic data (anteroposterior diameter and Palov ratio), and complications (axial symptoms and C5 palsy) were evaluated and compared among the groups.

There were 38, 40, and 27 patients in group A, B, and C, respectively. The overall facet joints destroyed rate was 30.7%. All groups gained significant JOA and NDI scores improvement postoperatively. The preoperative JOA, VAS, NDI scores, and postoperative JOA scores did not differ significantly among the groups. The group C recorded significant higher postoperative VAS scores than group A (*P* = 0.002) and B (*P* = 0.014) and had significant higher postoperative NDI scores than group A (*P* = 0.002). The pre- and postoperative radiologic data were not significant different among the groups. The group C had a significant higher axial symptoms incidence than group A (12/27 vs 8/38, *P* = 0.041).

Facet joints disturbance caused by miniscrews in plated cervical laminoplasty may not influence neurological recovery and spinal canal expansion, but may negatively affect postoperative axial symptoms.

## 1 Introduction

Cervical facet joints are synovial joints of the cervical spine which help maintain cervical spine stability and their destruction may disrupt proper kinetic balance.^[1,2]^ Of importance by analogy, injury and degeneration of these facet joints are also known to play important roles in many chronic cervical and shoulder pain syndromes.^[3,4]^ A total of 40% to 60% cases of chronic neck pain have been reported to be related to facet joints.^[3–5]^ Bykowski and Wong^[4]^ have previously reported that having greater than or equal to 3 facet joints involved may be more dangerous to have facet joint originated axial pain.

Plated or plate-only cervical laminoplasty has become an increasingly common technique for treating multilevel cervical spondylotic myelopathy.^[6–10]^ The plated cervical laminoplasty has the advantages like maintaining opened lamina position rigidly, preventing lamina reclosure, preserving more cervical range of motion (ROM) and cervical curvature, allowing early return to cervical exercise, and promoting hinge bony fusion compared to classic cervical laminoplasty.^[11,12]^ In plated cervical laminoplasty, miniplates were anchored to lateral masses and opened laminae by miniscrews. However, the complications associated with internal fixation in plated cervical laminoplasty are rarely discussed in literatures.

**Figure 1:**
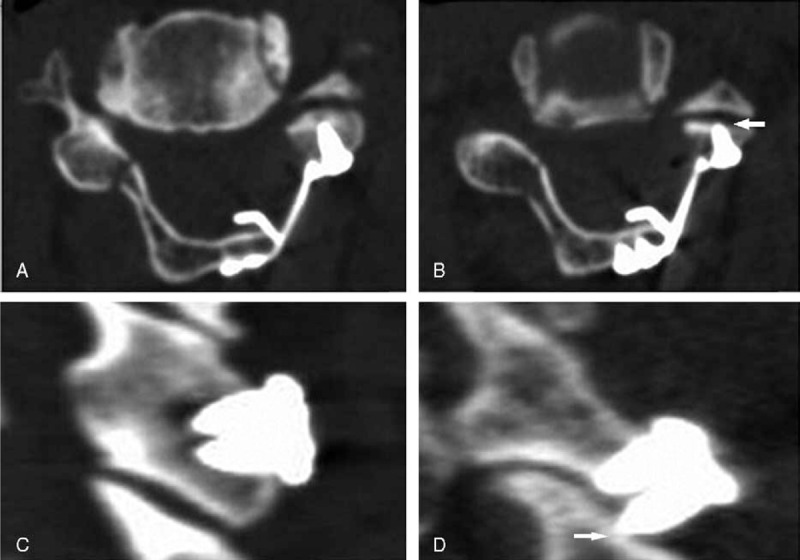
The identification of facet joints disturbance caused by miniscrews. (A, B) The axial CT images; (C, D) the sagittal CT images. (A, C) The screws did not penetrate into the facet joints; (B, D) the screws penetrated into the facet joints (the arrows).

The investigators have observed a unique facet joint disturbance caused by miniscrews where the miniscrews were used to anchor the miniplate to lateral mass that may allow them to penetrate the facet joint surface (Fig. 1). Although the facet joints are crucial of cervical spine, to the best of our knowledge, this facet joints disturbance has not been previously reported and it is, hitherto, unknown the incidence of this disturbance and whether this facet joint disturbance could impact clinical results following plated cervical laminoplasty. In the present study, we intend to investigate the clinical and radiologic data of patients with facet joints disturbance caused by miniscrews and to elucidate their potential role in plated cervical laminoplasty.

## 2 Materials and methods

The study cohort was approved by the Ethical Committee of West China Hospital of Sichuan University. All the patients had signed the informed consent form to allow their information to be used for research purposes. The patients who underwent C3 to C7 unilateral cervical laminoplasty with the Centerpiece miniplate fixation system (Centerpiece^TM^ Plate Fixation System; Medtronic SofamorDanek, Minnesota, USA) in the hospital between May 2010 and February 2014 were included in this study. And the patients who had received suture suspensory fixation, skipped miniplate fixation, suffered from unrelated preoperative neck and shoulder pain, had a history of sudden spinal injury, the operative levels were not C3 to C7, or underwent revision surgery were excluded. Patients would be divided into 3 groups according to the number of facet joints destroyed by miniscrews referred to Bykowski and Wong^[4]^: group A (none facet joint was destroyed), group B (1–2 facet joints were destroyed), and group C (3 or more than 3 facet joints were destroyed).

### 2.1 Surgical technique

After receiving general endotracheal anesthesia, the patient was positioned prone using a Mayfield 3-pin head-holder. A midline cervical incision was made to expose the laminae, spinous processes, and medial facet joints from C2 to C7. The ligaments were cut between C2 and C3 and between C7 and T1. The spinous processes were then amputated at their bases from C3 to C7. On the open side, a trough was created by completely cutting the lamina with a burr along the junction of the lateral mass and lamina. Once the open side was completed, an incomplete fracture hinge was created by making another trough on the opposing side. The lamina was carefully opened and an appropriately sized miniplate was inserted by fitting the cut edge of the lamina into the laminar shelf of the plate, then seating the lateral portion of the plate down onto the edge of the lateral mass. Two 7-mm miniscrews were used to anchor the plate to the lateral mass and two 5-mm miniscrews were inserted to anchor the plate to the opened lamina.

### 2.2 Clinical and radiologic evaluation

Evaluation of neurological function was performed before surgery and 1 week, 3 months, 6 months, 1 year, 3 years, and 5 years after surgery. The neurological function was assessed using the Japanese Orthopedic Association (JOA) score, and JOA recovery rates were calculated according to the formula: recovery rate = (JOA score after surgery − JOA score before surgery)/(17-JOA score before surgery) ∗ 100%.^[13]^ The complications including axial symptoms and C5 palsy were recorded. Axial symptom was defined as newly developed postoperative pain and stiffness anywhere from the nuchal to the scapular region which persisted for more than 3 months after laminoplasty.^[14]^ C5 palsy was defined as paresis of deltoid muscle developed after cervical spine surgery with no deterioration of myelopathy.^[15]^ The Visual Analogue Score (VAS) and The Neck Disability Index (NDI) were used to evaluate axial pain and daily activities involving the neck.

Radiologic evaluation included the X-ray films and computed tomography (CT) scans. Both of them were performed before the surgery. Meanwhile the X-ray films were performed at every follow-up time as a basic evaluation but the CT scans were performed only 3 and 6 months follow-up in order to observe hinge bony fusion. The anteroposterior diameter at C5 level, cervical alignment (C_2_–C_7_ angle), and cervical ROM were measured using the X-ray films, and Pavlov ratios were calculated.^[16]^ CT scans obtained at 3 and 6 months follow-up visits were used to evaluate the hinge bony fusion of each lamina. Bony fusion was determined to have occurred only when the dorsal and ventral cortices of the ends of the hinge fracture were completely fused together.^[8]^ Both sagittal and axial CT images obtained at 1 week postoperatively were used to assess the integrity of the facet joints. If the miniscrews that were used to anchor the miniplate to the lateral mass penetrated the facet joints, the facet joint was determined to have been destroyed.

### 2.3 Statistical analysis

Statistical analysis was performed using SPSS version 19.0 software (SPSS Inc., Chicago, IL). Continuous variables were presented as mean ± SD. The Student *t* tests were used to evaluate the differences of pre- and postoperative data when the data were normally distributed, and the Wilcoxon rank-sum test would be used when the data were not normally distributed. The 1-way ANOVA tests and Chi-square tests were used to evaluate the difference among the 3 groups. The missing data would not be included in statistical analysis. A *P*-value of less than 0.05 was considered statistically significant.

## 3 Results

### 3.1 Generally data

**Figure 2:**
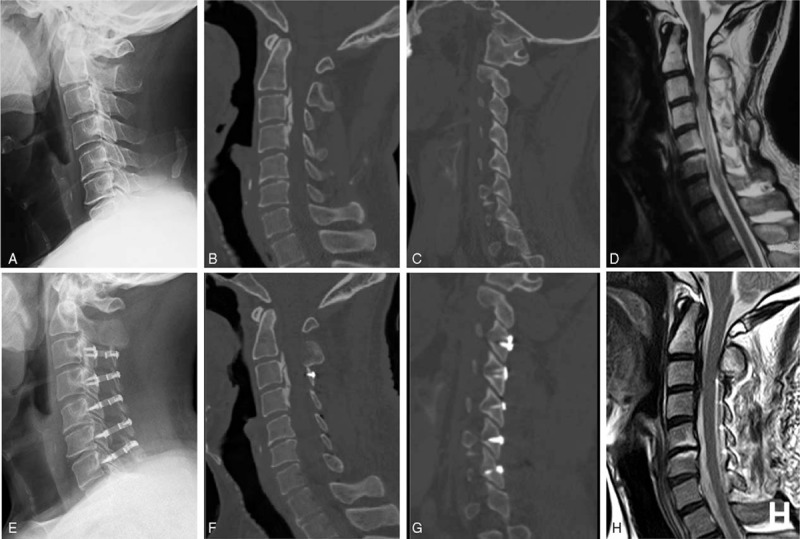
A 53-year-old male patients in group A. (A–D) Preoperative radiologic images. (E–H) Postoperative radiologic images. (A, E) Lateral X-ray film. (B, F) Middle sagittal CT reconstruction image. (C, G) The sagittal CT reconstruction image through facet joints on open side. (D, G) Midsagittal MRI image. CT = computed tomography, MRI = magnetic resonance imaging.

**Figure 3:**
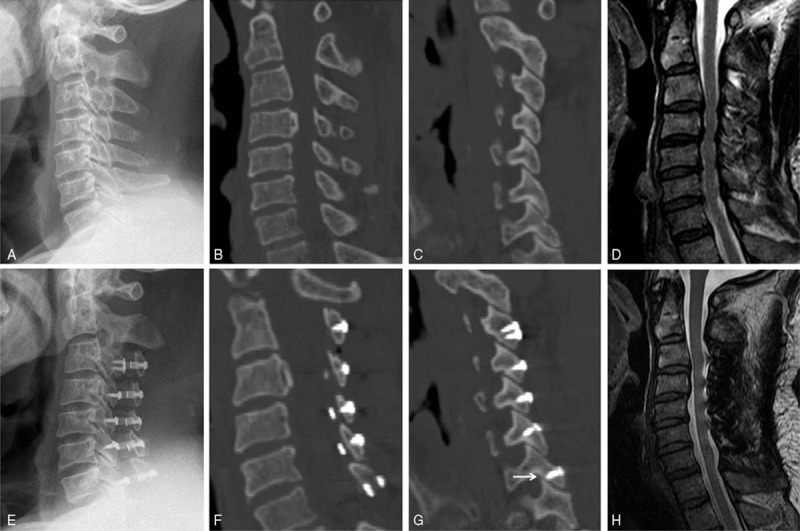
A 61-year-old male patients in group B. (A–D) Preoperative radiologic images. (E–H) Postoperative radiologic images. (A, E) Lateral X-ray film. (B, F) Middle sagittal CT reconstruction image. (C, G) The sagittal CT reconstruction image through facet joints on open side. There was 1 facet joint were destroyed by the miniscrew penetrating the facet joint (the arrow). (D, G) Midsagittal MRI image. CT = computed tomography, MRI = magnetic resonance imaging.

**Figure 4:**
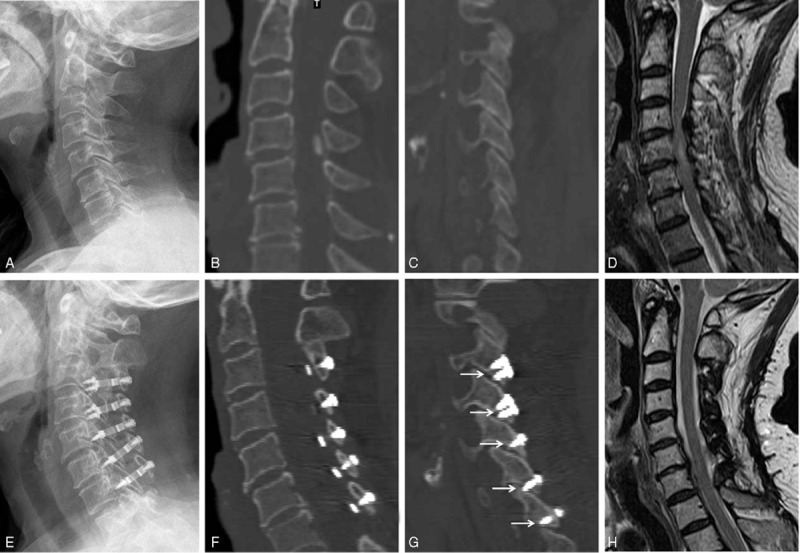
A 70-year-old female patients in group C. (A–D) Preoperative radiologic images. (E–H) Postoperative radiologic images. (A, E) Lateral X-ray film. (B, F) Middle sagittal CT reconstruction image. (C, G) The sagittal CT reconstruction image through facet joints on open side. There were 5 facet joints were destroyed by the miniscrews penetrating the facet joint (the arrow). (D, G) Midsagittal MRI image. CT = computed tomography, MRI = magnetic resonance imaging.

A total of 105 patients comprised of 84 males and 21 females with a mean age of 61 (range 31–89) years were included in the study. The average duration of symptoms was 22 (range 1–120) months. The operating time was 133 ± 21 minutes, blood loss was 271 ± 143 mL, and mean follow-up time was 45 (range 24–69) months. The operative levels involved C3 to C7 in 99 patients, C3 to C6 in 4 patients, C4 to C7 in 1 patient, and C2 to C7 in 1 patient. There were total of 521 laminae were fixed by miniplates. There were 160 facet joints destroyed by miniscrews on the open side, as determined by radiologic assessment. The facet joints destroyed rate was 30.7%. None facet joint for 38 patients, 1 facet joint for 19 patients, 2 facet joints for 21 patients, 3 facet joints for 15 patients, 4 facet joints for 6 patients, and 5 facet joints for 6 patients were destroyed. There were 38 patients in group A (Fig. 2), 40 patients in group B (Fig. 3), and the other 27 patients in groups C (Fig. 4). The gender, age, diagnose type, duration of symptoms, medical comorbidity, blood loss, operative time, and follow-up time did not differ significantly among the three groups (Table 1).

**Table 1:**
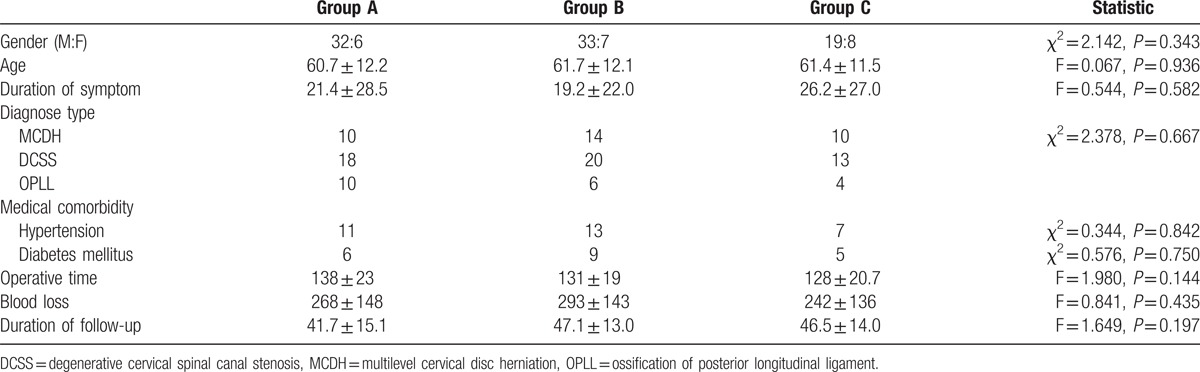
General data for the 3 groups.

### 3.2 Clinical outcomes

**Figure 5:**
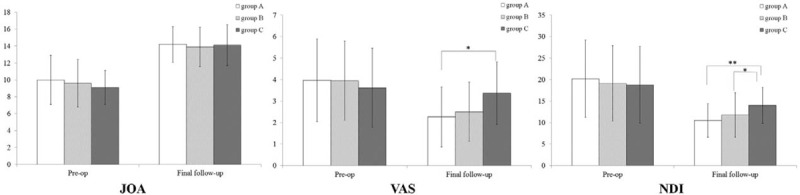
Comparison of JOA, VAS, and NDI scores among the 3 groups. ^∗^*P* < 0.05; ^∗∗^*P* < 0.01. JOA = Japanese Orthopedic Association, NDI = Neck Disability Index, VAS = Visual Analogue Score.

The overall JOA scores were 9.6 ± 2.7 before the surgery and 14.0 ± 2.2 at the final follow-up. The difference was significant. The recovery rate was 60.2% ± 21.9%. All 3 groups had significant JOA score improvement postoperatively (*P* < 0.05). There were no significant differences in preoperative JOA scores, postoperative JOA scores, and recovery rate among the 3 groups (*P* > 0.05). The preoperative VAS and NDI scores were not significant difference among the 3 groups. The VAS scores decreased significantly after the surgery for group A and B, but not for group C. The NDI decreased significantly after the surgery for all 3 groups (*P* < 0.05). The group C recorded significant higher postoperative VAS scores than group A (*P* = 0.002) and B (*P* = 0.014). The group C had significant higher postoperative NDI scores than group A (*P* = 0.002) (Fig. 5).

Axial symptoms were recorded in 32 patients. Eight of them were in group A, 12 of them were in group B, and 12 of them were in group C. The group C had a significant higher axial symptoms incidence than group A (*P* = 0.041). Four patients in group A, 6 patients in group B, and 3 patients in group C suffered C5 palsy after the surgery. Three patients in group A, 3 patients in group B, and 1 patient in group C had cerebrospinal fluid (CSF) leakage after the surgery. The difference of C5 palsy and CSF leakage among the groups were not significant different (*P* > 0.05).

### 3.3 Radiologic outcomes

**Figure 6:**
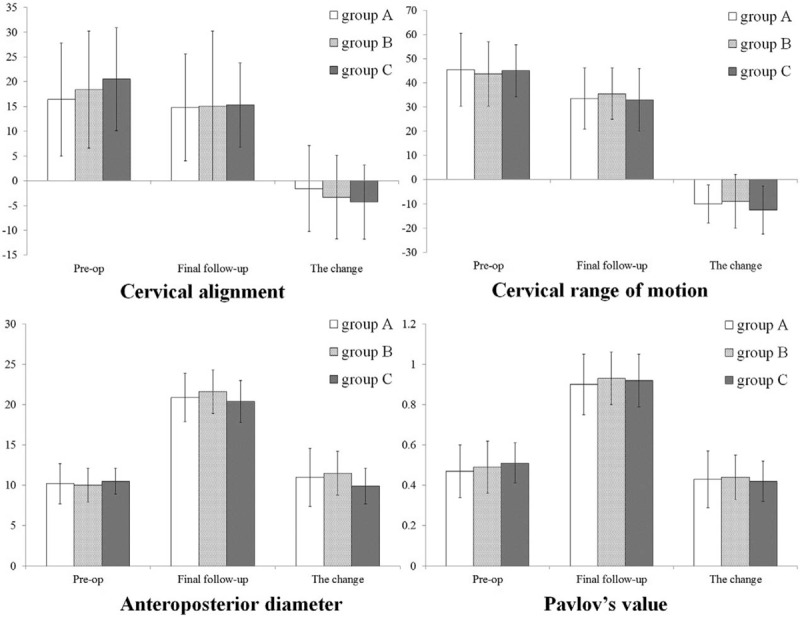
Comparison of radiologic data among the 3 groups.

Cervical alignment, AP diameter, Pavlov value, and cervical ROM were significantly different before and after surgery for all groups. These pre- and postoperative radiologic data and their changes among the groups were not statistically significant different (Fig. 6). CT scan data were available for all patients at 1-week postoperative. Eighty eight patients had CT scans data at 3 month follow-up and 79 patients had CT scans data at 6 month follow-up. The hinge union rate was 73.9%, 68.1%, and 71.1% for group A, B, and C at 3 months postoperative. It was 88.3%, 90.3%, and 94.0% for group A, B, and C at 6 months postoperative. The differences of hinge union rate at 3 and 6 months were not significant among the groups (*P* > 0.05). One lamina miniscrew was back-out during the follow-up. No laminae reclosure, no plate dislodged or broken, and no spinal cord or nerve root injury by the miniscrews was observed during the follow-up.

## 4 Discussion

The plated cervical laminoplasty technique has recently become an increasingly popular method to treat multilevel cervical spondylotic myelopathy since first introduced by O’Brien in 1996.^[10]^ The miniplate fixation system may offer an immediate, rigid fixation for the laminae and stabilize the cervical spinal canal expansion.^[7]^ However, the investigators noticed a unique facet joints disturbance on the open side that is easily overlooked in plated cervical laimnoplasty. The miniscrews used to anchor the miniplate to the lateral mass may penetrate the facet joint surface, and, indeed, our findings indicate that this kind of facet joint injury was not an uncommon event. There was 30.7% destroyed facet joints attributable to miniscrews within the patients of our cohort and 25.7% (27/105) patients had 3 or more than 3 facet joints destroyed.

Facet joints are important posterior spine structure. The 2 facet joints make contributions to cervical stability and make up the constrained 3 balance points together with the intervertebral disc during spine finishing movements.^[1,2]^ The injury or the mechanical changes of the facet joints can alter the structural integrity and articular cartilage health which can potentially lead to degeneration and painful arthritic changes.^[4]^ The roles of facet joints were discussed in many cervical spine surgeries. Li et al^[17]^ reported that the facet joints load increase in spinal intervertebral fusion and suggested this may be the initial factor of adjacent segment degeneration. Jaumard et al^[18]^ measured the facet joint load change after total disc replacement and reported potential complications of facet arthrosis at follow-up. In classic cervical laminoplasty using suture suspensory method to fix laminae, suturing and stretching of the facet joints capsule, dissection around the facet joints, and postsurgical facet scarring were considered as important sources of postoperative axial symptoms.^[6,12,14,19]^ Chen et al^[12]^ concluded that it is better to keep the facet joint intact and reduce the invasion of facet joints in cervical laminoplasty.^[12,19]^

In the present study, we divided the patients into 3 groups according to different number of facet joints destroyed referred to previous study. We found that different groups had similar neurologic improvement and spinal canal enlargement. Although group A and C recorded higher postoperative JOA scores compared to group B, the differences were not significant. These results indicated that the iatrogenic facet joints destroyed by laminoplasty lateral mass miniscrews may not influent the decompression effect of the surgery and interrupt the neurologic recovery.

Axial pain is one of the most common and serious complication after cervical laminoiplasty.^[20,21]^ Plated cervical laminoplasty limits facet joints suture damage that is commonly seen in classic laminoplasty which has the potential to induce axial pain. Chen et al^[12]^ hypothesized that this may be the reason why plated cervical laminoplasty was associated with a lower rate of axial symptoms in their study. In the present study, the overall axial pain rate was 30.5% (32/105), little lower than the axial pain rates reported by Yeh et al^[6]^ and Jiang et al.^[22]^ Several measures had been done during the treatment including protection of facet joint capsule during preparing the open and hinge trough, carefully separate and reconstruct the paraspinal muscle, and early cervical spine rehabilitation guidance. All these may help us to gain a relatively lower axial pain rate.

**Figure 7:**
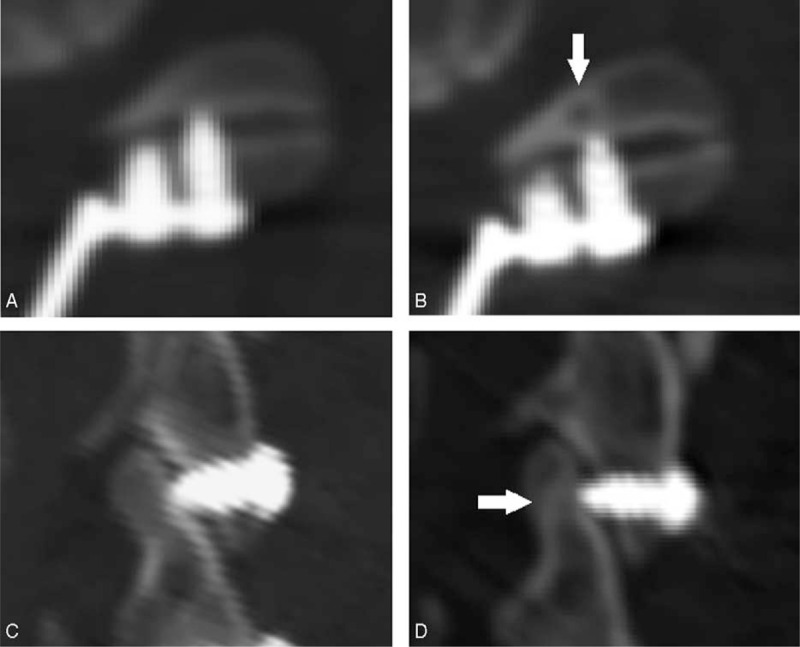
Facet joint osteoarthritis after the facet joint destroyed. (A, B) The axial CT images. (C, D) The sagittal CT images. (A, C) At 1 week postoperative, the facet joint was destroyed by the miniscrew and no osteoarthritis existed. (B, D) Two years follow-up, the facet joint osteoarthritis formed, osteolysis and osteosclerosis existed (the arrow).

Meanwhile, in the present study, the group C had much higher axial pain rate than group A and the patients in group C recorded significant higher VAS scores as well as NDI scores than the patients in group A. These results suggest that the facet joints induce by the miniscrews may have negative effect on postoperative axial pain. As the facet joint is a synovial joint and is important for cervical stability and mobilization, the miniscrew penetration of the articular surfaces may injure the synovial villi of the articular surfaces, negatively impact cervical stability, induce sterile inflammation, and lead to facet joint degeneration or osteoarthritis (Fig. 7).^[2,4]^ All these may contribute to the persistence of the neck pain. Taken together, facet joints disturbance caused by miniscrews may be potential risk factors of postoperative axial pain. We also found that patients with more facet joints destroyed by miniscrews suffered more serious pain, suggesting a potential “dose–response” relationship. These findings are in keeping with Bykowski conclusions.^[4]^ Thus, cervical surgeons should be aware of the importance of this potential risk and carefully operate to protect the facet joints during surgery. Park and Heller^[23]^ had suggested to place the miniplate on the superior portion of the lateral mass to avoid penetration of the miniscrews into the facet joint. We also recommended that the miniscrews should be implanted more on the upper portion of the lateral mass, and short miniscrews might be better for small lateral mass.

However, the underlying causes of axial pain have not been fully clarified yet. Beside the facet joint disturbance, the destruction of posterior cervical structures, cervical spinal nerve root damage, cervical lordosis and rang of cervical movement decrease, and dystrophy of the posterior muscles after surgery are reported to be related to axial pain.^[14,20,24]^ A multivariate analysis may be necessary to help identify the confounding factors and risk factors of axial pain after plated cervical laminoplasty in future study.

Other complications after cervical laminoplasty including C5 palsy and CSF leakage were not significant difference among the 3 groups. There was also no spinal cord or nerve root injury observed after surgery in our study. Different from the lateral mass screws used in posterior cervical spine fusion, the miniscrews used in the present study are much shorter (5–7 mm).^[9]^ It may be safe to implant the miniscrews without spinal cord or nerve root injury. Our study also showed that the facet joints disturbance on open side may not influence the hinge bony fusion.

Some limitations exist in our study. The sample size was small and the axial pain incidence was relative low in the present study. The complex mechanisms of axial pains, the low axial pain rate, and the small sample size of our study may affect the statistical validity. The large range of follow-up time may also result in some bias of postoperative evaluation. Another limitation was that the lack of long-term follow-up CT scan data which failed us to analysis facet joint fusion effect of miniscrews penetrating facet articular surface. The ignorance of the high risk and potential outcomes of this complication and the inappropriate miniscrews implant techniques may be the causes of this facet joints disturbance. A proper miniscrew implant method could be discussed in future study.

In conclusion, our study retrospectively reported the clinical and radiologic outcomes of the patients with facet joints destroyed by lateral mass miniscrews in plated cervical laminoplasty. We found that though facet joints disturbance may not influence postoperative neurological recovery and radiologic improvement, but may negatively affect the postoperative axial pain following plated cervical laminoplasty. Our findings emphasize the importance of the facet joints and the surgeons should be carefully operative to avoid facet joints damage in the surgery.
